# Accreditation Council for Graduate Medical Education Milestone Training Ratings and Surgeons’ Early Outcomes

**DOI:** 10.1001/jamasurg.2024.0040

**Published:** 2024-03-13

**Authors:** Brigitte K. Smith, Kenji Yamazaki, Ara Tekian, Benjamin S. Brooke, Erica L. Mitchell, Yoon Soo Park, Eric S. Holmboe, Stanley J. Hamstra

**Affiliations:** 1 Accreditation Council for Graduate Medical Education, Chicago, Illinois; 2 Department of Medical Education, University of Illinois College of Medicine, Chicago; 3 Division of Vascular Surgery, Department of Surgery, School of Medicine, University of Utah, Salt Lake City; 4 University of Tennessee Health & Science Center, Memphis; 5 Department of Surgery, University of Toronto, Toronto, Ontario, Canada; 6 Holland Bone and Joint Program, Sunnybrook Research Institute, Toronto, Ontario, Canada; 7 Department of Medical Education, Northwestern University Feinberg School of Medicine, Chicago, Illinois

## Abstract

**Importance:**

National data on the development of competence during training have been reported using the Accreditation Council for Graduate Medical Education (ACGME) Milestones system. It is now possible to consider longitudinal analyses that link Milestone ratings during training to patient outcomes data of recent graduates.

**Objective:**

To evaluate the association of in-training ACGME Milestone ratings in a surgical specialty with subsequent complication rates following a commonly performed operation, endovascular aortic aneurysm repair (EVAR).

**Design, Setting, and Participants:**

This study of patient outcomes followed EVAR in the Vascular Quality Initiative (VQI) registry (4213 admissions from 208 hospitals treated by 327 surgeons). All surgeons included in this study graduated from ACGME-accredited training programs from 2015 through 2019 and had Milestone ratings 6 months prior to graduation. Data were analyzed from December 1, 2021, through September 15, 2023. Because Milestone ratings can vary with program, they were corrected for program effect using a deviation score from the program mean.

**Exposure:**

Milestone ratings assigned to individual trainees 6 months prior to graduation, based on judgments of surgical competence.

**Main Outcomes and Measures:**

Surgical complications following EVAR for patients treated by recent graduates during the index hospitalization, obtained using the nationwide Society for Vascular Surgery Patient Safety Organization’s VQI registry, which includes 929 participating centers in 49 US states.

**Results:**

The study included outcomes for 4213 patients (mean [SD] age, 73.25 [8.74] years; 3379 male participants [80.2%]). Postoperative complications included 9.5% major (400 of 4213 cases) and 30.2% minor (1274 of 4213 cases) complications. After adjusting for patient risk factors and site of training, a significant association was identified between individual Milestone ratings of surgical trainees and major complications in early surgical practice in programs with lower mean Milestone ratings (odds ratio, 0.50; 95% CI; 0.27-0.95).

**Conclusions and Relevance:**

In this study, Milestone assessments of surgical trainees were associated with subsequent clinical outcomes in their early career. Although these findings represent one surgical specialty, they suggest Milestone ratings can be used in any specialty to identify trainees at risk for future adverse patient outcomes when applying the same theory and methodology. Milestones data should inform data-driven educational interventions and trainee remediation to optimize future patient outcomes.

## Key Points

**Question:**

Does assessment of surgical competence using Accreditation Council for Graduate Medical Education Milestones during training predict future patient outcomes?

**Findings:**

In this study, Milestone ratings near the end of surgical training were associated with complication rates in patients treated by those surgeons early in their practice.

**Meaning:**

Milestone ratings may help programs to identify specific gaps in competence during training that could be remediated prior to graduation.

## Teaser

This study evaluates the association of in-training Accreditation Council for Graduate Medical Education Milestone ratings in a surgical specialty with subsequent complication rates following a commonly performed operation, endovascular aortic aneurysm repair.

## Introduction

In 2013, the Accreditation Council for Graduate Medical Education (ACGME) implemented a requirement for all training programs to report Milestone ratings for each trainee.^1^ This mandate was prompted by concerns about variations in quality of care and especially the prevalence of medical errors.^2^ It was hypothesized that variations in patient outcomes among early-career physicians were linked to significant variability in training.^3,4^ The Milestones were intended to identify and correct for this variability, thus, ultimately improving patient care.^4^ Despite this initiative, program directors, institutional officials, and trainees remain concerned about the enormous cost and burden of the Milestones, in light of the fact that there is limited empirical evidence of effectiveness in improving the quality of resident education.^5,6,7,8^

A key advance would be linking performance during training to patient outcomes following training. A landmark general surgery study by Birkmeyer et al^9^ demonstrated a direct link between individual surgeon technical skill and complication rates. A subsequent study^10^ linked site of training and obstetrician complication rates following graduation. Research is needed that combines both of these approaches to understand whether a specialist’s individual skills acquired during training can be linked to subsequent performance and outcomes following graduation. The purpose of this study was to evaluate the association of national ACGME Milestone ratings with postoperative complication rates for surgeons during their early career.

## Methods

We sought to determine whether Milestone ratings during vascular surgery training were associated with complications following endovascular aortic aneurysm repair (EVAR) performed by recent graduates of vascular surgery training programs. EVAR is a commonly performed, complex procedure conducted to treat a life-threatening condition in high-risk patients. We linked individual vascular surgeons’ ACGME Milestone ratings from residency and fellowship training to EVAR patient outcomes, collected as part of the Society for Vascular Surgery Patient Safety Organization’s Vascular Quality Initiative (VQI) registry after those surgeons entered independent practice. The study was designated nonhuman participants research by the University of Utah institutional review board. The requirement for informed consent was waived.

### Cohort Definition

**Figure 1.**
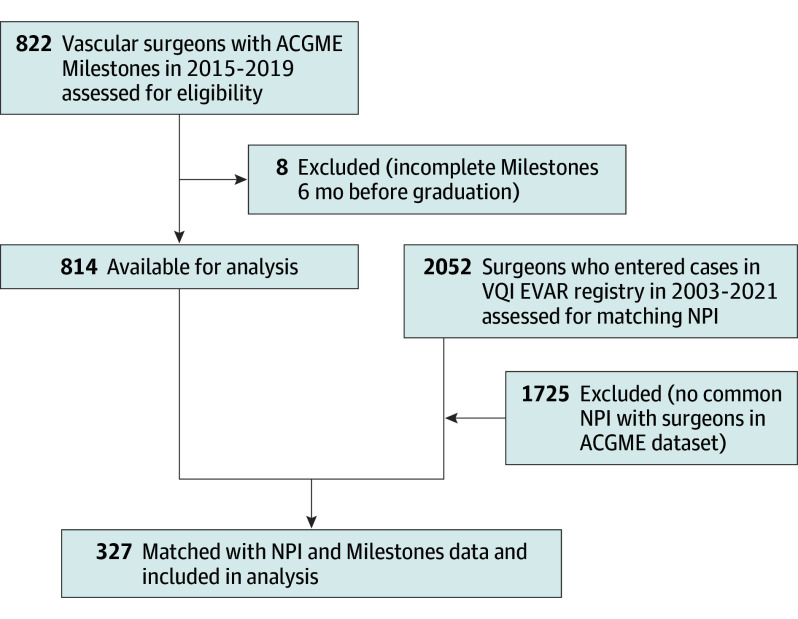
Surgeon Cohort Definition Assembly of cohort for analysis. ACGME indicates Accreditation Council for Graduate Medical Education; EVAR, endovascular aortic aneurysm repair; NPI, national provider identifier; VQI, Vascular Quality Initiative.

The surgeon cohort definition is outlined in Figure 1. We used the ACGME national database to identify all graduates of vascular surgery training programs in the US between 2015 and 2019 (2015 was the first year of ACGME Milestones data reporting). We included graduates of both vascular surgery fellowships and integrated vascular surgery residency programs, as both of these paradigms result in vascular surgery board eligibility and include training in how to perform EVAR. Vascular surgeons were included in the study if they had complete ACGME Milestones data at 6 months prior to graduation. Surgeons were excluded if they did not have Milestones data available or if they did not enter cases into the VQI EVAR registry.

### Exposure Variable—Measures of Surgeon Competence: ACGME Milestones

Milestone ratings are reported to the ACGME by individual training programs in each specialty. The ACGME Milestones describe an individual trainee’s development as a physician within the 6 core competencies of patient care, medical knowledge, systems-based practice (SBP), practice-based learning and improvement, professionalism, and interpersonal and communication skills.^11,12^ The introduction of Milestones as a competency rating system during residency and fellowship has shown many process benefits in terms of educational practice, but there is not yet any research showing their predictive power to explain variation in subsequent clinical practice.^13^ The Vascular Surgery Milestones include 31 subcompetencies. Each subcompetency is rated on a 5-point scale with level 4 categorized as ready for unsupervised practice.

### Delphi Process

To determine a priori conceptual alignment between Milestone subcompetencies and patient outcomes following EVAR, we gathered expert consensus using a Delphi process.^14^ This resulted in 15 of 31 subcompetencies for vascular surgery deemed to be most relevant for predicting complications following EVAR (5 patient care, 7 medical knowledge, 1 systems-based practice, 2 interpersonal and communication skills). These 15 subcompetencies were used to construct a composite Milestone rating. The remaining 16 subcompetencies were excluded from analysis.

### Main Outcome Measures—Complications Following EVAR

The primary study outcomes were major and minor complications during the index hospitalization following EVAR. Major complications included a composite of intraoperative kidney artery coverage, conversion to open repair, postoperative intestinal ischemia, major adverse cardiac event, new dialysis, respiratory failure, stroke, reoperation, and death. Minor complications included a composite of access site complications, distal embolism requiring intervention, iliac artery injury, iliofemoral thrombectomy, intraoperative endoleak, and postoperative change in kidney function.

### Description of VQI

All patient outcome data during the study time period were obtained using the VQI quality registry, established in 2011, which currently includes 929 participating centers in 49 US states.^15,16^ VQI prospectively collects patient-, surgeon-, and hospital-level data, and reports risk-adjusted postoperative outcomes for vascular surgical procedures, both during the index hospitalization, as well as long-term follow-up. The VQI includes 14 distinct clinical registries, each representing a category of vascular procedures. Patient comorbidities and procedure-specific postoperative complications are captured. Outcome data are collected for every eligible procedure and for every procedural surgeon, for a minimum of 1-year postprocedure.

### Statistical Analysis

The present study focused on the association between surgeons’ Milestone ratings during training and patient outcomes following EVAR in their early career, accounting for the differences in patients’ and surgeons’ characteristics. The matched dataset was analyzed for surgeon performance, controlling for patient-level and surgeon-level covariates, using a 2-level model (ie, patients nested within surgeons at a particular hospital). Each of the major and minor complications following EVAR was regressed on control variables and aggregate Milestone ratings for each individual, using a generalized estimating equations (GEE) logistic model. Patient-level (age, sex, functional status, and unfit for open abdominal aortic aneurysm [AAA] repair) and surgeon-level (case volume) covariates were selected for adjustment in the model based on established risk factors for complications following EVAR. To account for the fact that patient outcomes were structured in a 2-level hierarchy (ie, patients nested within surgeon-hospital), correlations among outcome observations were specified using a standard exchangeable working correlation matrix in the GEE model, assuming that any 2 patient outcomes within a surgeon-hospital have the same correlations. The GEE analyses controlled for patient age, gender, functional status (1 = fully functional, light work; 0 = self-care, assisted care, bed bound), presurgical determination of whether the patient was unfit for open AAA repair, and surgeon’s case volume (as a measure of surgeon experience).

To clarify the interpretation of regression coefficients for the composite Milestones rating as a predictor, it was decomposed into the residency program mean and each trainee’s deviation from the mean, based on previous research showing substantial program-level effects in the Milestone ratings, consistent with multilevel analyses.^17,18,19,20,21^ This led to the inclusion of an interaction term which highlights the conditional effect of Milestone ratings by program. All statistical analyses were performed using SAS Enterprise Guide, version 7.15 (SAS Institute).

## Results

ACGME Milestones data were available for 822 vascular surgeons who completed training from 2015 through 2019. Surgeons missing Milestones assessments 6 months prior to graduation were excluded (n = 8). ACGME Milestones data were linked to surgeon-specific clinical data from the VQI EVAR registry using national provider identification numbers, yielding 327 (40.2% match) early-career vascular surgeons practicing at 208 VQI-participating centers (Figure 1). Among these surgeons, 4213 EVARs were completed from 2015 through 2021. The mean (SD) and median case volume for the 327 surgeons were 12.88 (14.58) and 8.00, respectively, ranging from 1 to 99. The 487 surgeons not included in the analysis did not have their national provider identification registered in VQI, meaning they worked in centers not participating in the registry.

The rate of complications was 9.5% for major (400 of 4213 cases) and 30.2% for minor (1274 of 4213 cases) complications. The mean (SD) age of patients was 73.25 (8.74) years and most were male with good functional status (Table 1).

**Table 1.** Patient Demographics

Demographic	No. (%)
Age, y, mean (SD)	73.25 (8.74)
Gender	
Female	834 (19.8)
Male	3379 (80.2)
Unfit for open AAA repair	
Yes	690 (16.4)
No	3459 (82.1)
Missing	64 (1.5)
Full functional status^a^	
Yes	3447 (81.8)
No	626 (14.9)
Missing	140 (3.3)

Abbreviation: AAA, abdominal aortic aneurysm.

^a^ The Vascular Quality Initiative defines full functional status as ability to do light housework. Non–full functional status is defined as requiring assisted care.

Mean composite Milestone ratings for the 327 surgeons 6 months prior to graduation was 4.03 (0.44), ranging from 2.53 to 5.00. The program-level means of composite Milestone ratings ranged from 2.99 to 4.97 with a mean (SD) of 3.97 (0.34) across 102 unique sponsoring institutions (ie, each health care sponsoring institution oversees graduate medical education [GME] training in 1 or more specialties, consisting of different unique training programs). The program-centered individual composite Milestone deviation ratings ranged from −1.23 to 1.39 with a mean (SD) of 0.05 (0.34) for the surgeons included in the analyses (Table 2).

**Table 2.** Exposure Variables

Variable	No.	Mean	SD	Minimum	25th Percentile	Median	75th Percentile	Maxium
Composite Milestone rating^a^	327	4.03	0.44	2.53	3.77	4.00	4.30	5.00
Deviation of surgeon Milestone rating from program Milestone mean	327	0.05	0.34	−1.23	−0.14	0.09	0.25	1.39
Program Milestone mean	102	3.97	0.34	2.99	3.77	3.94	4.21	4.97

^a^ Average of Milestone ratings on 15 subcompetencies identified as relevant to patient outcomes following endovascular aortic aneurysm repair.

For all outcomes, older and female patients who were categorized as unfit for open AAA repair tended to be associated with increased rates of complications than their counterparts. Every unit increase in surgeons’ case volume was associated with decreased complications; the association was significant for major complications only (odds ratio [OR], 0.99; 95% CI, 0.98-1.00; Table 3).

**Table 3.** Odds Ratios (OR) of the Logistic Regression Model

Characteristic	Major complications, OR (95% CI)	*P* value	Minor complications, OR (95% CI)	*P* value
Age (increasing)	1.01 (1.00-1.02)	.05	1.018 (1.01-1.03)	<.001
Female gender (binary; affirmative)	1.35 (1.06-1.72)	.02	1.31 (1.12-1.53)	.001
Unfit for open AAA repair (binary; affirmative)	1.76 (1.37-2.26)	<.001	1.28 (1.04-1.58)	.02
Full functional status (binary; affirmative)^a^	0.81 (0.62-1.06)	.13	1.09 (0.90-1.34)	.40
Surgeon case volume (No. of cases; increasing)	0.99 (0.98-1.00)	.01	1.00 (1.00-1.01)	1.00
Deviation of surgeon Milestone rating from program Milestone mean (A) (1.0 Milestone rating scale increment; increasing)^b^	0.50 (0.26-0.95)	.03	0.731 (0.45-1.18)	1.00
Program Milestone mean (B) (1.0 Milestone rating scale increment; increasing)	0.82 (0.53-1.27)	.40	0.74 (0.54-1.01)	.01
Interaction between A and B	3.35 (1.03-10.92)	.05	3.73 (1.53-9.10)	.001

Abbreviation: AAA, abdominal aortic aneurysm.

^a^ The Vascular Quality Initiative defines full functional status as ability to do light housework. Non–full functional status is defined as requiring assisted care.

^b^ Main effect calculated when program Milestone mean was set to 3.50.

**Figure 2.**
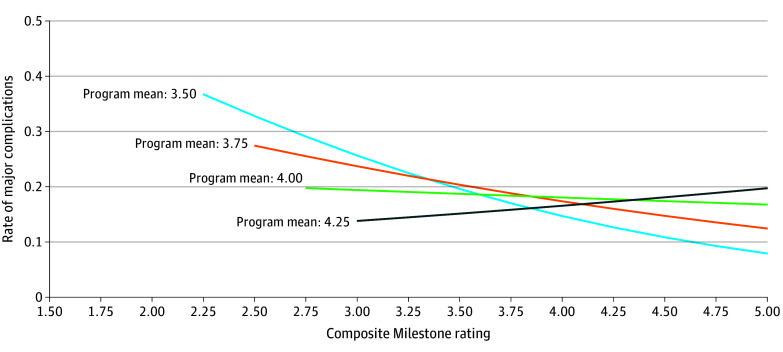
Accreditation Council for Graduate Medical Education Milestone Ratings and Major Complications Following Endovascular Aortic Aneurysm Repair For graduates of training programs with Milestone mean ratings of 3.50, the odds ratio (OR) of surgeons’ risk of major complications was 0.50, which is equivalent to an increase in risk by 2.00 times for every 1.0-point decrease in Milestone ratings (95% CI, 0.26-0.95). Program means were 3.75 (OR, 0.68; 95% CI, 0.44-1.03); 4.00 (OR, 0.92; 95% CI, 0.65-1.29); and 4.25 (OR, 1.24, 95% CI, 0.77-2.00), respectively.

Results indicate a significant interaction effect between program-level Milestone mean and trainee-specific deviations from the program-level Milestone mean (Table 3). This interaction effect indicates that, depending on site of training (ie, program), there was a significant association between individual Milestone ratings of surgical trainees 6 months prior to graduation and complications in early career (interaction term for major complication: OR, 3.35; 95% CI, 1.03-10.92; minor complication: OR, 3.73; 95% CI, 1.53-9.10; Table 3). For surgeons who graduated from programs with lower mean Milestone ratings, the association with complication rates was strong, but this effect was much weaker for graduates from programs with higher mean Milestone ratings (Figure 2). An example derived from the results in Table 3 demonstrates the association between predicted rates of major complications following EVAR and Milestone ratings by program means for a typical patient, where the typical patient is male, 73 years old, unfit for open AAA with nonfunctional status, and has been treated by a surgeon with a case volume of 8. The difference in shapes of the 4 curves illustrates the interaction effect (Figure 2). Close examination of Figure 2 shows that the associations between major complication rates and Milestone ratings varied as program mean changed, meaning that surgeons with higher ratings than their counterparts within the same program tended to be associated with decreased complications, but this association was localized only for those programs with lower program-level mean Milestone ratings. For graduates of training programs with Milestone mean ratings of 3.50, the OR of surgeons’ risk of major complications was 0.50, which is equivalent to an increase in risk by 2.00 times for every 1.0-point decrease in Milestone ratings (OR, 0.50; 95% CI, 0.26-0.95). The odds of risk increase were not statistically significant for training programs with other Milestone mean ratings: for program mean = 3.75: (OR, 0.68; 95% CI, 0.44-1.03); 4.00 (OR, 0.92; 95% CI, 0.65-1.29]); and 4.25: (OR, 1.24; 95% CI, 0.77-2.00).

## Discussion

The ability to predict patient outcomes from differences in learner ability during training is arguably the most important goal in GME. With the new mandate for competency-based medical education and its focus on frequent assessment of trainees, the size and scope of the resulting national database shows—the first time—the association of detailed longitudinal data on resident performance during training with patient outcomes following training.

Our findings suggest that national ACGME Milestone ratings of graduating vascular surgeons are may be predictive of those surgeons’ risk-adjusted patient outcomes in their early career following a common vascular operation. Furthermore, the risk of future complications is directly associated with trainees’ Milestone rating deviation from their respective program mean rating. To our knowledge, this is the first study to demonstrate a significant association between comprehensive assessments of surgeon competence during training (ACGME Milestones) and patient care outcomes in clinical practice following graduation.

Prior research on this topic using the national Milestones database is limited to just one study, in which no link was found between Milestone ratings during training and subsequent measures of patient outcomes, as captured by Medicare claims data.^22^ There are several notable differences between this prior work and the current study. First, the vascular surgery Milestones provide greater specificity within the competency domains of patient care and medical knowledge than the general surgery Milestones. The Milestones reporting form includes language that differentiates between open and endovascular technical skills; explicitly identifies basic, intermediate, and advanced procedures; and enables the selection of subcompetencies that are most relevant to specific procedures and outcomes, which we identified in previous work through a Delphi process.^14^ Second, the VQI registry provides an outcomes dataset that is uniquely suited to supporting this line of inquiry through collection of procedure-specific outcomes and comprehensive case capture. Lastly, we controlled for program effects, recognizing that rater behavior could result in systematic bias of ratings of individual trainees’ performance, depending on the program in which they train.

Most other work on correlations with Milestones data has focused on board examination performance and certification status as the measure of physician competence. Findings have been mixed depending on the specialty and outcomes database used with roughly half of studies demonstrating no association.^23^ Importantly, even for studies with positive findings, board certification occurs after completion of training, making it too late for use as a mechanism to identify physicians requiring remediation in the supervised training environment.^24,25,26^ The ACGME Milestones address these longstanding shortcomings of GME assessment systems by providing detailed and specific feedback in the form of roughly 22 Milestones subcompetency sets per specialty (range, 12-41).

### Implications for Training

The ACGME Milestones reporting system was created in response to a recognized need for GME systems to have greater accountability to the public with regard to the competence of graduates as part of the transition to outcomes-based medical education.^3^ Longitudinal assessment data captured throughout training and across competency domains hold great promise to enable early detection of underperforming trainees and provide specific recommendations for remediation at actionable time points.^27,28,29,30,31^

The current study indicates that Milestones assessments of surgeon competence are able to detect trainees who are more likely to underperform in practice. This follows from previous work on the ability of early Milestone ratings to predict Milestone ratings at graduation.^30^ The utility of the Milestone ratings for identifying vascular surgeons who may struggle with higher complication rates following EVAR was highest in those training programs with lower Milestone mean ratings. One possible explanation is that such programs may have been wary of grade inflation and understand better the heuristic value of using the entire range of Milestone ratings,^5,32^ as systematic differences in Milestone implementation processes between programs have been reported.^7,8^ Regardless, vascular surgery programs can use the results of this study to identify trainees needing additional educational interventions. For example, if a resident has low Milestone ratings with 18 months left in training, the program director would be well-advised to intervene. Training programs can also leverage the predictive probability values provided by the ACGME to identify struggling trainees earlier.^33^

### Implications for Patients

These findings have important implications for patients. The GME system, including accrediting and certifying bodies, as well as individual training programs, can begin to use Milestones assessment data to identify trainees who are at risk of having poor patient outcomes after graduation. This aligns with the intended formative purposes of the Milestones to use outcomes research as feedback to training programs for the purpose of continuous quality improvement. By remediating deficiencies in a supervised environment, patient safety is protected and outcomes can be optimized.

### Realizing the Potential of Milestones

Our findings have major implications for the GME system. ACGME Milestones are used by every medical specialty and every ACGME-accredited GME program in the US. Evidence that achievements within the Milestones assessment system are predictive of patient care outcomes in clinical practice strengthens the case for programs to continue to collect Milestones data with as much rigor as possible and provides validity evidence to further justify the use of these ratings during training.^21^ The results of this research could be used to set achievement benchmarks for competency-based advancement throughout residency and fellowship training, identify struggling trainees at time points enabling effective coaching, and support establishment of achievement standards for graduation to ensure physician competence and satisfactory patient care outcomes in independent clinical practice.^34^ In addition, training programs could use the results presented here to identify and address educational gaps in their curriculum.

The ACGME national Milestones database is now mature enough to compare it with patient registry-based outcomes data. The implications for graduate medical education are significant. Milestone ratings could be used to predict future adverse patient outcomes and identify trainees in need of remediation while still in the supervised environment. In principle, these methods are generalizable beyond vascular surgery to any specialty. We see this work following from the landmark article by Birkmeyer et al^9^ in general surgery demonstrating a direct link between individual surgeon (ie, not trainees) technical skill and complication rates, and the larger and more comprehensive analysis by Asch et al^10^ that demonstrated a linkage between site of training (ie, not individuals) and obstetrician complication rates.

### Limitations

Our findings should be considered in the context of several limitations. First, our study population represents only vascular surgeons practicing at VQI-participating hospitals, which could impact generalizability of the findings. Nevertheless, the VQI includes hospitals in nearly every US state across all geographic regions, and prior research has demonstrated no significant differences in process measures associated with quality of care delivery at VQI and non–VQI participating hospitals.^35^ Second, as vascular surgery represents a relatively small specialty, generalizability of the findings to other specialties may be limited. Third, while the results concerning the associative value of Milestone ratings were statistically significant, the 95% CIs were quite large, indicating substantial imprecision for predicting future complication rates. Lastly, in constructing the composite Milestone ratings used in this study, we used a Delphi process involving content experts^14^ that involved equal weighting of the subcompetencies and it may be that this equal weighting may not reflect a consensus of the vascular surgery community at large.

## Conclusions

Physician competence is essential to delivering high-quality health care, as reflected by patient outcomes. GME programs should demonstrate that meeting defined measures of competence, assessed during training, is correlated with patient outcomes in unsupervised practice after training. This correlation is needed to identify factors that may predict poor clinical performance before entering independent practice, enabling GME systems to remediate deficiencies among trainees before they graduate. The ability to harness and use measures of physician competence to predict future patient outcomes is a powerful contribution to ensuring patient safety.

## Notes

Supplement. Data sharing statement
