# Supplementary material for: Accreditation Council for Graduate Medical Education Milestone Training Ratings and Surgeons’ Early Outcomes
Source: JAMA Surg. 2024 Mar 13;159(5):546–52. doi: 10.1001/jamasurg.2024.0040 (PMC10938242; doi:10.1001/jamasurg.2024.0040)
Supplement: Supplement. — Data sharing statement [file jamasurg-e240040-s001.pdf]

Data Sharing Statement

Smith. Accreditation Council for Graduate Medical Education Milestone Training Ratings and Surgeons’ Early Outcomes. *JAMA Surg*. Published March 13, 2024.
doi:10.1001/jamasurg.2024.0040

Data
Data available: No

Additional Information
Explanation for why data not available: PHI data will not be publicly available as patient data part of a registry; however, Milestone data is available.
